# Retraction: Underwater image enhancement using multi-task fusion

**DOI:** 10.1371/journal.pone.0360350

**Published:** 2026-10-12

**Authors:** The PLOS One Editors

After this article [1] was published, similarities were noted between this article and previously published work by different authors [2], which call into question the validity and provenance of the reported results. In light of these issues, the *PLOS One* Editors retract this article [1].

All authors either did not respond directly or could not be reached.
